# 2388. Factors Associated with COVID-19 Vaccination Among Racial/Ethnic Minority Populations with HIV, Miami-Dade County, Florida, 2022

**DOI:** 10.1093/ofid/ofad500.2008

**Published:** 2023-11-27

**Authors:** Mary Jo Trepka, Daisy Ramirez-Ortiz, Michele Jean-Gilles, Robert Ladner, Tan Li, Diana Sheehan

**Affiliations:** Florida International University, Miami, Florida; Florida International University, Miami, Florida; Florida International University, Miami, Florida; Behavioral Science Research Corporation, Coral Gables, Florida; Florida International University, Miami, Florida; Florida International University, Miami, Florida

## Abstract

**Background:**

People with HIV (PWH) are at increased risk for COVID-19 complications and thus can significantly benefit from COVID-19 vaccination. PWH are also more likely to belong to racial/ethnic minority groups that have been underrepresented among vaccinated individuals. The objective of this analysis was to identify Health Belief Model (e.g., perceived susceptibility to and severity of COVID-19, perceived barriers, and benefits of COVID-19 vaccination) and Social Ecological Model (i.e., intrapersonal, interpersonal, and community/institutional-level) factors associated with vaccine uptake among PWH.

**Methods:**

The cross-sectional survey was administered by telephone in English, Spanish or Haitian Creole from January–March 2022 to a sample of 299 adult PWH receiving medical case management services through the Miami-Dade County Ryan White Program (MDCRWP). Participants who received a primary vaccine series were classified as fully vaccinated, and those that received only 1 dose of a 2-dose vaccine series or no doses were classified as not fully vaccinated. Multivariable logistic regression was used to estimate adjusted odds ratios (aORs) of full vaccination with 95% confidence intervals (CIs). The final adjusted model included all sociodemographic characteristics selected a priori, as well as covariates with a p-value < 0.2 in bivariate analyses. Analyses were weighted to be representative of the race/ethnicity and sex distribution of clients in the MDCRWP.

**Results:**

The weighted percentages for full vaccination was 83.8% for the entire group, and 88.9% among Hispanics, 72.0% among African Americans, and 67.5% among Haitians. Being fully vaccinated was associated with Hispanic compared with African American race/ethnicity (aOR 5.44; 95% CI 1.50-19.77), not endorsing any misconceptions about COVID-19 vaccines (aOR 8.26; 95% CI 1.38-49.64), reporting encouragement to get vaccinated from their sources of information (aOR 20.82; 95% CI 5.84-74.14), and perception that ≥ 50% of social network was vaccinated (aOR 3.35; 95% CI 1.04-10.71) (Table 1).

**Figure:**
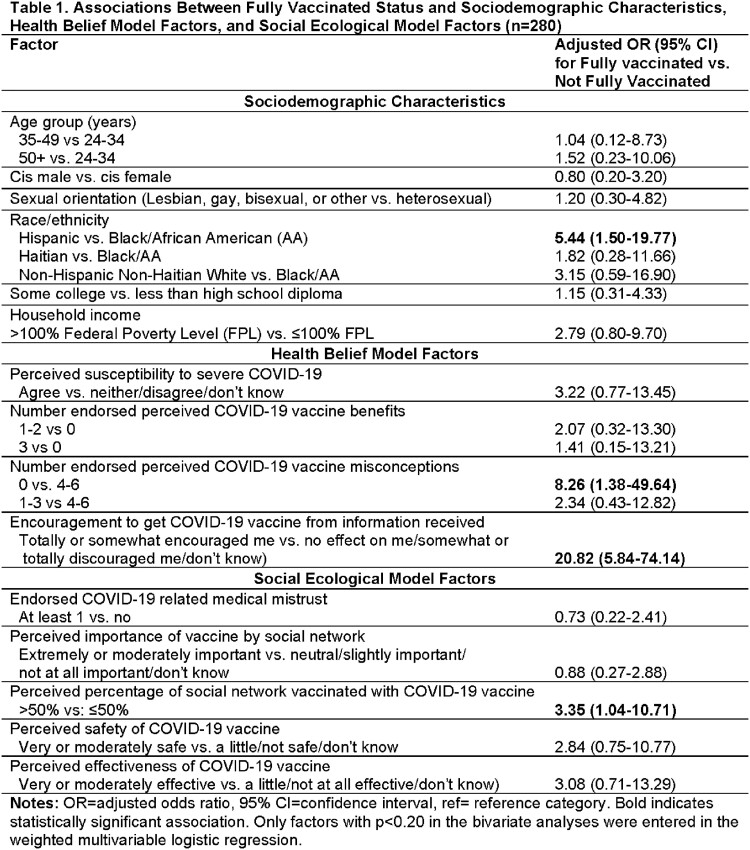

**Conclusion:**

Results highlight the importance of promoting accurate information from sources deemed trustworthy, and social networks in COVID-19 vaccine uptake among PWH.

**Disclosures:**

**All Authors**: No reported disclosures

